# Large-Scaled Metabolic Profiling of Human Dermal Fibroblasts Derived from Pseudoxanthoma Elasticum Patients and Healthy Controls

**DOI:** 10.1371/journal.pone.0108336

**Published:** 2014-09-29

**Authors:** Patricia Kuzaj, Joachim Kuhn, Ryan D. Michalek, Edward D. Karoly, Isabel Faust, Mareike Dabisch-Ruthe, Cornelius Knabbe, Doris Hendig

**Affiliations:** 1 Institut für Laboratoriums- und Transfusionsmedizin, Herz- und Diabeteszentrum Nordrhein-Westfalen, Universitätsklinik der Ruhr-Universität Bochum, Bad Oeynhausen, Germany; 2 Metabolon, Inc., Durham, North Carolina, United States of America; University Hospital S. Maria della Misericordia, Udine, Italy

## Abstract

Mutations in the ABC transporter ABCC6 were recently identified as cause of Pseudoxanthoma elasticum (PXE), a rare genetic disorder characterized by progressive mineralization of elastic fibers. We used an untargeted metabolic approach to identify biochemical differences between human dermal fibroblasts from healthy controls and PXE patients in an attempt to find a link between ABCC6 deficiency, cellular metabolic alterations and disease pathogenesis. 358 compounds were identified by mass spectrometry covering lipids, amino acids, peptides, carbohydrates, nucleotides, vitamins and cofactors, xenobiotics and energy metabolites. We found substantial differences in glycerophospholipid composition, leucine dipeptides, and polypeptides as well as alterations in pantothenate and guanine metabolism to be significantly associated with PXE pathogenesis. These findings can be linked to extracellular matrix remodeling and increased oxidative stress, which reflect characteristic hallmarks of PXE. Our study could facilitate a better understanding of biochemical pathways involved in soft tissue mineralization.

## Introduction

Pseudoxanthoma elasticum (PXE) is a heritable disease arising from mutations in the ABC transporter ABCC6 and is characterized by soft tissue calcification and fragmentation manifested in the skin, eyes and cardiovascular system [1]. The progressive mineralization of the elastic fibers is accompanied by remodeling of the extracellular matrix (ECM) [2] and enhanced circulating levels of matrix metalloproteinases (MMPs) [3]. Furthermore, yellow papular lesions and wrinkling of the skin at flexural body sites are apparent characteristics of PXE [4]. The retina of PXE patients is affected by calcification of the Bruch's membrane, with pigment modifications (peau d'orange) and fractures (angioid streaks), whereupon recurring neovascularization, hemorrhages and cicatrization can lead to central vision loss [5]. Progressive calcifications of the arterial walls can lead to cardiovascular dysfunction characterized by decreased peripheral pulses, claudication, hypertension, or coronary-artery disease with angina and/or myocardial infarcts [6]. The prevalence of the autosomal recessive disease PXE is estimated to be between 1∶25.000 and 1∶100.000 [4], and to date up to 350 ABCC6 mutations were described [7] with p.R1141X (20–30%) and c.EX23_EX29del (5–15%) being the most frequent in European PXE patients [8].

Although ABCC6 is primarily expressed in the liver where the transporter is located at the basolateral membrane of the hepatocytes, gene expression studies demonstrate low levels in kidney, intestine [9], and human dermal fibroblasts [10]. Until now, no causal link between the mutations in ABCC6 and soft tissue calcification in PXE has been found. Clinical manifestations with aberrant mineralization affect peripheral tissues like skin, eyes, kidney, or blood vessels in PXE [5]. Today, two models are discussed for explaining the putative pathomechanisms in PXE, the “metabolic” and the “peripheral cell” hypotheses.

The “metabolic hypothesis” tries to explain the role of ABCC6 in the liver as a supplier of an/or several unknown substrate/s to the whole-body circulation [11], [12]. Surprisingly, the liver itself is not affected in the majority of PXE patients [5]. Transport studies for ABCC6 revealed that glutathione-conjugates like glutathione S-conjugated leukotriene C4 (LTC4), N-ethylmaleimide S-glutathione (NEM-GS), and S-(2,4-dinitrophenyl) glutathione could be transported *in vitro* [13], [14]. To explore function and substrate specifications of ABC transporters, recent strategies have involved proteomics [15], targeted metabolomics [16], [17] or transportomic approaches as used recently for ABCC2 (MRP2) [18]. Van *et al.* used 2D total correlation spectroscopy NMR to compare the global metabolic profiles of urine obtained from wild-type and Abcc6 knockout mice [19]. Indeed, Jansen *et al.* [20] investigated cells overexpressing ABCC6 by an untargeted metabolic approach analyzing 25 compounds. The authors reported an accumulation of nucleoside triphosphates in the supernatant of ABCC6-overexpressing HEK-293 cells. However, LC-MS analysis of control and Abcc6^−/−^ mouse plasma revealed no differences in these metabolites.

We describe a large untargeted metabolic approach identifying biochemical changes and differences between human dermal fibroblasts from healthy controls and PXE patients, searching for a causal link between ABCC6 mutations, cellular metabolic alterations and disease pathogenesis. Our cell culture model implemented a 24-h cultivation period without supplementation of fetal calf serum (FCS) to avoid potential metabolite interference, affecting ABCC6 gene expression or transport activities. Without external supplementation, cells become constrained for the intracellular synthesis of essential metabolites, whereupon potential substrates of ABCC6 might accumulate in the cytosol of fibroblasts derived from PXE patients. Serum starvation was further conducted to induce oxidative stress conditions in human dermal fibroblasts [21], as well as to enhance metabolic and proteolytic activity in these cells [22], to closely mimic the described cellular phenotype of PXE. Additionally, serum withdrawal was shown to induce ABCC6 mRNA expression significantly, compared to standard cultivation method in 10% FCS [23].

## Materials and Methods

### Cell culture

Dermal fibroblasts from six healthy controls (NHDF) were purchased from Promocell (Heidelberg, Germany), Genlantis (San Diego, USA), Cambrex (Walkersville, USA) and Coriell (New Jersey, USA). Dermal fibroblasts from six PXE patients were received as described before [10] as well as from Coriell (New Jersey, USA). All cells were isolated and characterized by standard methods. The study was approved by the ethics commission of the Ruhr University of Bochum Faculty of Medicine, located in Bad Oeynhausen. All patients provided their written informed consent to participate in the study. Cell lines and characteristics are summarized in Table S1. Fibroblasts were cultivated from passages 6–10, ensuring comparibility in gender and age. Cells were cultivated in a CO_2_ incubator at 37°C using 100-mm cell culture dishes (Greiner bio-one, Frickenhausen, Germany) containing 10 ml Dulbecco's modified essential medium (DMEM; Gibco) with 10% fetal calf serum, 1% L-glutamine (200 mM) and 1% antibiotic/antimycotic solution (PAA, Pasching, Austria). For experiments cells were grown for 24 h in 10% FCS (177 cells/mm^2^; BD Falcon), washed twice with phosphate-buffered saline (PBS; Gibco), and replaced with DMEM without FCS for additional 24 h. Cells reached approx. 70–80% of confluence after stated time of growth. Five biological samples were prepared as replicates for metabolomics studies, as well as further triplicates for gene and protein expression analysis.

Gene-silencing of ABCC6 was carried out using Lipofectamine 2000 reagent (Invitrogen- Life technologies, Darmstadt, Germany). ABCC6-specific small-interfering RNA (siABCC6) (siRNA-ID 106395) and FAM labeled control siRNA oligonucleotides (siNK) (Ambion- Life technologies, Darmstadt, Germany) were delivered to dermal fibroblasts of healthy controls during reverse transfection, in a total siRNA-concentration of 40 nM. No antibiotic/antimycotic solution was used during siRNA experiments for the first 12 h. Cell culture medium was replaced with fresh media 12 h post-transfection, whereupon transfection efficiencies were examined by fluorescence microscopy of FAM labeled controls. Cells were cultivated for additionally 24 h in 10% FCS, followed by 24 h cultivation without FCS.

After stated time of growth, cells were washed with PBS, detached by trypsinization and pelleted by centrifugation (3 min, 1000×g), whereupon the cell pellet was flash-frozen in liquid nitrogen and stored at −80°C until mass spectrometric analysis was performed.

Cells harvested for gene expression analysis were detached by trypsinization and pelleted by centrifugation (3 min, 1300×g). Pellets were washed with PBS and centrifuged again, whereupon residues were resuspended in lysis buffer RA1 (Macherey–Nagel, Düren, Germany). Nucleic acid extraction and reverse transcription were carried out as described previously [24], at which 2 µg RNA were used for cDNA synthesis. Real-Time quantitative PCR (qPCR) was performed using LightCycler 480 and LightCycler 480 SYBR Green I Master reaction mixture (Roche, Penzberg, Germany). Primer sequences for reference genes (ACTB, GAPDH, ß2M) and ABCC6 were used as published before [10].

### Protein extraction and Western Blotting

For the preparation of protein lysates cells were washed twice with ice cold PBS and harvested by scraping into a microcentrifuge tube. Cells were pelleted by centrifugation (5 min, 3000×g), whereupon the cell pellet was resuspended in 200 µl lysis buffer, containing 137.5 mM NaCl, 50 mM Tris/HCl (pH 7.8), 8.7% Glycerin, 0.5 mM EDTA (pH 8.0), 1% Protease Inhibitor (P2714; Sigma Aldrich, Taufkirchen, Germany), 1% NP-40 (Sigma Aldrich, Taufkirchen, Germany). Cell lysates were frozen at −80°C, again clarified by centrifugation (10 min, 8000×g), aliquoted and stored at −80°C. Total protein content of cell lysates was estimated using bicinchoninic acid assay (BCA Kit, Sigma Aldrich, Taufkirchen, Germany).

Protein samples were separated by 8–16% Tris-Glycine gel with Tris-Glycine SDS running buffer (Novex, Life technologies, Darmstadt, Germany). Lysates were loaded at a concentration of 14.5 µg protein per well to the gel and separated with electrophoresis for 2 h. PVDF membranes were used for protein blotting (Life technologies, Darmstadt, Germany). For the detection of ABCC6, blots were incubated with primary (Anti-MRP6, ERP8342, rabbit monoclonal, 1∶5000; Anti-GAPDH, ab8245, mouse monoclonal, 1∶2000; abcam, Cambridge, England) and secondary antibodies (Goat poly secondary antibody to rabbit IgG- H+L, HRP, ab97051, 1∶2000; rabbit polyclonal secondary antibody to mouse IgG- H+L, HRP, ab6728, 1∶2000; abcam, Cambridge, England) in TBS-T buffer. Immunoreactivity was detected by chemiluminescence using AceGlow (PEQLAB Biotechnologie GMBH, Erlangen, Germany). Images and densitometric analysis were performed on Fusion-SL detection system (PEQLAB Biotechnologie GMBH, Erlangen, Germany).

### Metabolomic Analysis

Metabolomic profiling analysis was performed by Metabolon, Inc. as previously described [25], [26]. Detailed description of mass spectrometry analysis is given in supplemental material (Methods S1).

### Statistical analysis

Experimental data are indicated as means ± S.E. Graphic data processing and statistics were performed with GraphPad Prism 5 (GraphPad Software, Inc., La Jolla, USA) and Array Studio (Omicsoft Corp., Cary, USA) using student's t-test with Welch's correction (two tailed; confidence interval of 95%). Data analysis has been corrected by multiple testing with false discovery rate (FDR). PCA analysis was performed with Array Studio (Omicsoft Corp., Cary, USA).

## Results

**Figure 1:**
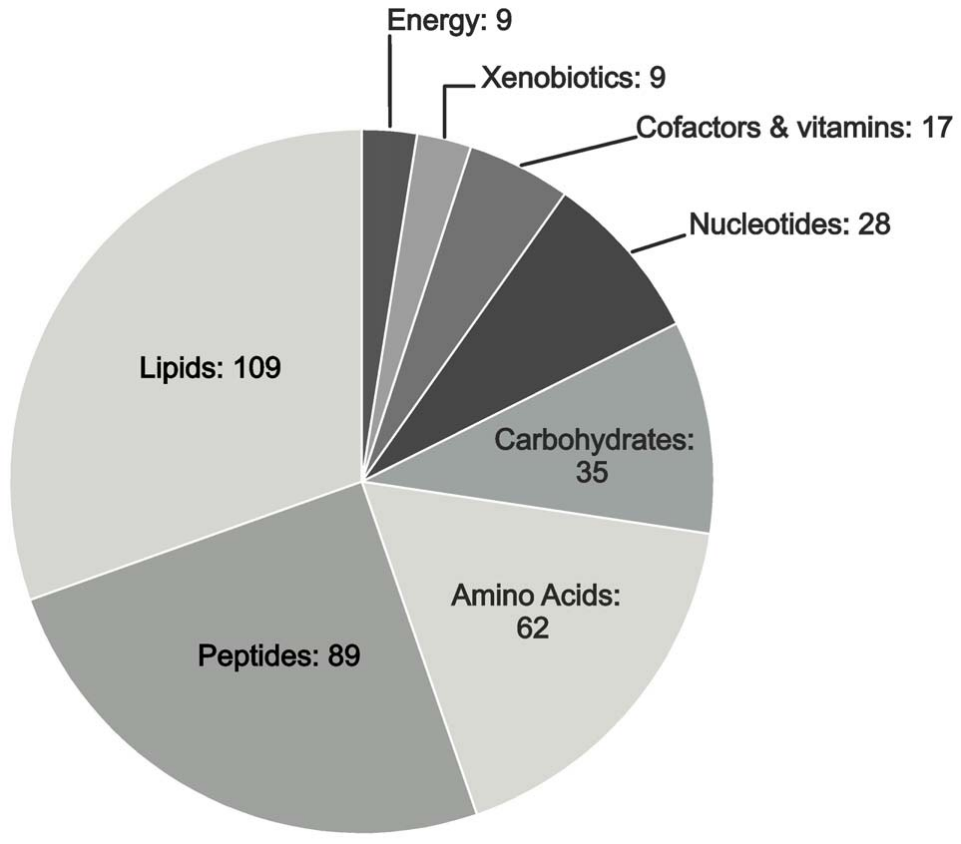
Gene expression and Western Blot analysis of ABCC6. (a) Quantification of ABCC6 mRNA expression in human dermal fibroblasts from healthy controls (n = 5; white) and PXE patients (n = 6; black). Effect of siRNA-mediated knockdown on ABCC6 gene expression: fibroblasts transfected with a scramble siRNA-negative control (siNK, n = 3; white); ABCC6-specific siRNA-treated cells (siABCC6, n = 3; black). Expression levels are normalized to reference gene expressions (ACTB, GAPDH, ß2M). Data are presented in arbitrary units as means with corresponding standard error. Differences between controls and PXE fibroblasts, as well as between siRNA-transfected cells, were analyzed using unpaired t-test with Welch's correction. (b) Western Blot analysis of ABCC6 in fibroblasts of healthy controls, PXE patients and siRNA- treated cells. Western blot analysis was performed with pooled protein samples from each group, with GAPDH as normalization control. HEK-293 cell lysates were used as an additional blotting control. (c) Representative Western Blot for fibroblasts of healthy controls, PXE patients and (d) siRNA- treated cells.

PXE, control fibroblasts and siRNA-transfected cells were classified by gene expression levels of ABCC6 and protein detection by Western Blot (Figure 1). No mRNA expression was found in PXE fibroblasts (Figure 1a); therefore C_t_ values were set to 35 for normalization. Gene expression of ABCC6 was down-regulated by up to 75% in cells treated with ABCC6-specific siRNA (Figure 1a). Statistical comparison of all groups was omitted due to variance in sample cohort size (controls, PXE n = 5–6 vers. siRNA-treated cells n = 3); comparisons were only made between controls and PXE cells, as well as between siRNA-transfected cells.

Protein analysis by Western Blot revealed an overall 30% reduced protein level of ABCC6 in PXE fibroblasts relative to controls (Figure 1b, c). PXE patient 2 exhibited the lowest ABCC6 content with about 28% that found in controls, whereas PXE patients 3, 5 and 6 showed ABCC6 levels between 50 and 69%. However, two samples (PXE 1 and 4) had similar ABCC6 expression to control cells. Furthermore, samples from ABCC6 knockdown experiments showed similar ABCC6 expression to scrambled negative siRNA controls (siNK) 48 h post-transfection (Figure 1b, d).

**Figure 2:**
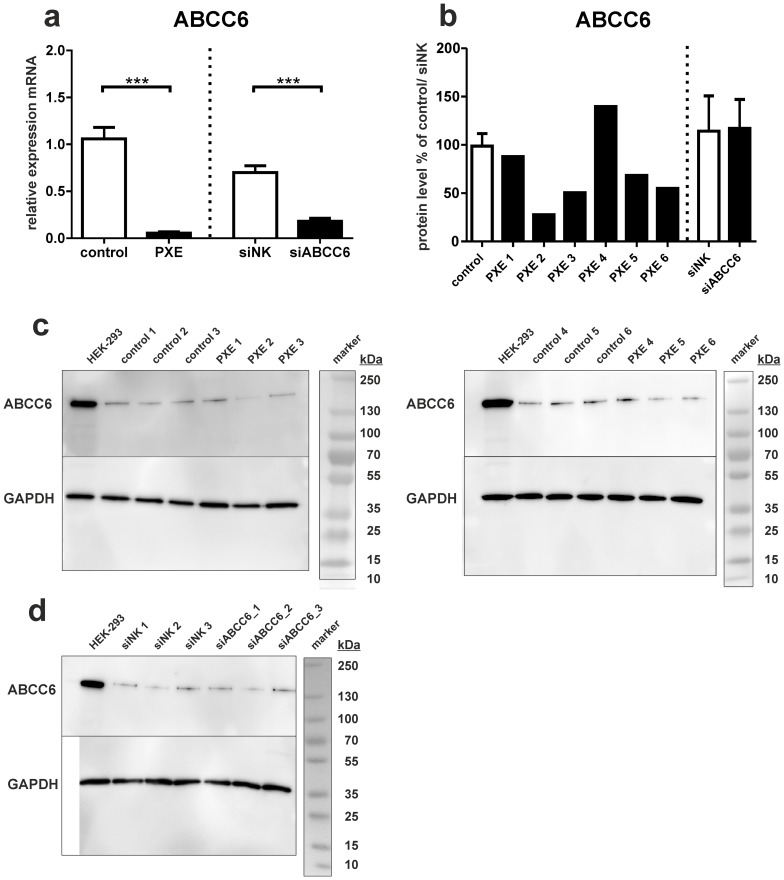
Metabolic profiling: Biochemical classes of analyzed metabolites.

Unbiased global biochemical profiling of PXE and control cells resulted in the detection of 358 named compounds encompassing lipids, amino acids, peptides, carbohydrates, nucleotides, vitamins and cofactors, xenobiotics and energy metabolites (e.g. citrate, pyrophosphate) as shown in Figure 2. The dominant compound group consisted of lipid metabolites, including essential-, long chain-, monohydroxy-, dicarboxylate-, and branched fatty acids, as well as prostaglandins, eicosanoids, carnitines, sphingolipids, steroids and phospholipids.

Metabolic profiles of human dermal fibroblasts of healthy controls and PXE patients, as well as siRNA-transfected cells were compared by principle component analysis (PCA, Figure S1); however, no general metabolic clustering between healthy controls and PXE patients or within transfected cells was found. Based on this overall matching, one cell line of the control cohort (Ctrl CL2) was omitted for further analysis due to principle deviation.

Overall, different compound abundances were found between PXE patients and controls, as well as for siRNA-transfected cells, which are summarized in Table S2 classified by biochemical pathways. Cell lysates of PXE patients showed 85 significantly elevated metabolites (p<0.05), and 21 compounds were significantly reduced in comparison to healthy controls. In contrast, compared to scrambled siRNA negative control (siNK), siRNA mediated loss of ABCC6 resulted in only 5 significantly increased and 67 diminished metabolites, suggesting a critical difference between these two model systems.

### Metabolic profiling of Fatty acids (FA) and Glycerophospholipids (GP)

**Figure 3:**
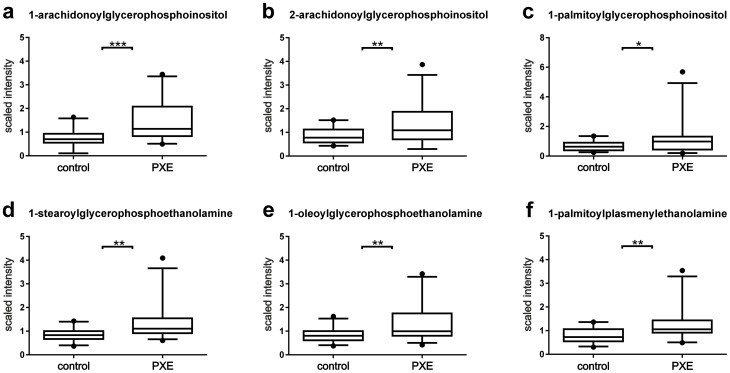
Analysis of Glycerophospholipids. Glycerophospholipids were detected by LC-MS/MS in negative ionization mode: (a) 1-arachidonoylglycerophosphoinositol (patient/control ratio:2.4, p<0.0001); (b) 2-arachidonoylglycerophosphoinositol (patient/control ratio:1.6, p<0.007); (c) 1-palmitoylglycerophosphoinositol (patient/control ratio:1.8, p<0.03); (d) 1-stearoylglycerophosphoethanolamine (patient/control ratio:1.6, p<0.003); (e) 1-oleoylglycerophosphoethanolamine (patient/control ratio:1.6, p<0.003); (f) plasmalogen 1-palmitoylplasmenylethanolamine (patient/control ratio 1.7, p<0.002).

Glycerophospholipids were analyzed by GC- and LC-MS/MS and classified into phosphatidylcholines (PC), phosphatidylethanolamines (PE) and phosphatidylinositols (PI). Multiple glycerophospholipids including 1-arachidonoylglycerophosphoinositol (patient/control ratio:2.4, p<0.0001), 2-arachidonoylglycerophosphoinositol (patient/control ratio:1.6, p<0.007), 1-palmitoylglycerophosphoinositol (patient/control ratio:1.8, p<0.03), 1-stearoylglycerophosphoethanolamine (patient/control ratio:1.6, p<0.003), 1-oleoylglycerophosphoethanolamine (patient/control ratio:1.6, p<0.003) and the plasmalogen 1-palmitoylplasmenylethanolamine (patient/control ratio:1.7, p<0.002), were significantly increased in PXE fibroblasts and are shown in Figure 3. Similarly, the essential fatty acids dihomo-linolenate (20:3n3 or n6), docosapentaenoate (22:5n3) and docosapentaenoate (22:5n6) accumulated at a significantly higher rate in PXE cells compared to controls (Figure S2; patient/control ratio:1.4, p<0.03; 1.5, p<0.0006; 1.3, p<0.05, respectively). Although other essential fatty acids were also augmented in PXE samples, the differences were not statistically significant (Table S2). In contrast to glycerophospholipids and essential fatty acids, long chain fatty acids did not differ between healthy controls and PXE patients, with the exception of arachidonate (20:4n6) and adrenate (22:4n6) which were elevated in PXE fibroblasts (Figure S2; patient/control ratio:1.5, p<0.005; 1.3, p<0.04, respectively). Additionally, the monohydroxy fatty acids 2-hydroxystearate and 2-hydroxypalmitate were diminished in cell lysates of PXE patients (patient/control ratio:0.7, p<0.05; 0.7, p<0.02, respectively), while4-hydroxybutyrate (GHB) accumulated (Table S2; patient/control ratio:1.5, p<0.001). Finally, increased levels of sphingosine and palmitoyl sphingomyelin (Figure S3; patient/control ratio:1.3, p<0.02; 1.3, p<0.0001, respectively), and elevated amounts of the cholesterol precursor lathosterol (Figure S3; patient/control ratio:1.4, p<0.05) were found in cell lysates of PXE fibroblasts. Due to the lipid-based siRNA delivery system (Lipofectamine), siRNA transfected cells were omitted in further lipid analysis (3.1.); complete information is given in Table S1.

### Leucine and Isoleucine dipeptides are increased in PXE fibroblasts

**Figure 4:**
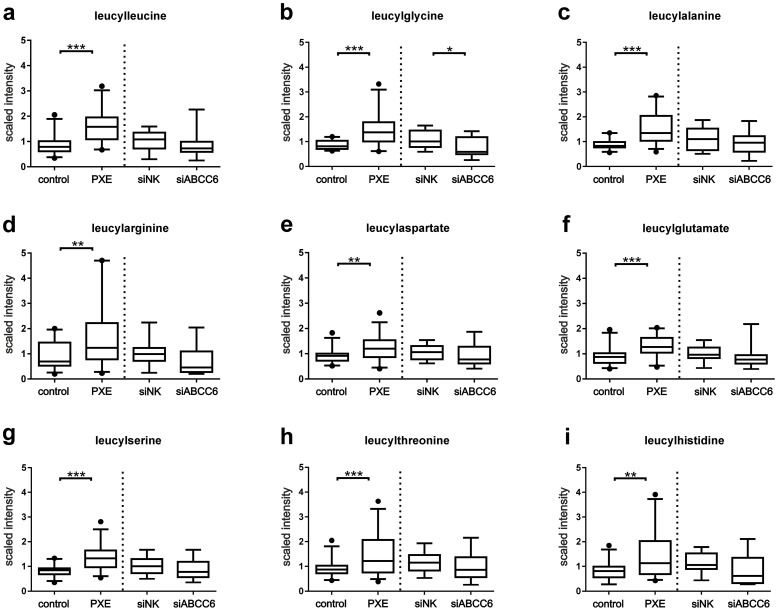
Leucyl dipeptides. Scaled intensities of Leucyl dipeptides were detected by LC-MS/MS positive/negative: (a) leucylleucine (patient/control ratio:1.9, p<0.0001), (b) leucylglycine (patient/control ratio:1.7, p<0.0001), (c) leucylalanine (patient/control ratio 1.7, p<0.0001), (d) leucylarginine (patient/control ratio:1.9, p<0.005), (e) leucylaspartate (patient/control ratio:1.4, p<0.002), (f) leucylglutamate (patient/control ratio:1.4, p<0.0004), (g) leucylserine (patient/control ratio:1.6, p<0.0001), (h) leucylthreonine (patient/control ratio:1.7, p<0.002), (i) leucylhistidine (patient/control ratio:1.8, p<0.002). No significant differences were observed between siRNA-transfected cells.

Peptides represented the second largest class of metabolites detected by metabolic profiling in this study (Figure 1). Of these biochemicals, mass spectrometric analysis identified 81 dipeptides with a marked difference in the accumulation of leucine peptides in PXE samples relative to healthy controls (Figure 4). Specifically, leucylleucine, leucylglycine, leucylalanine and leucylserine peptides were significantly elevated in PXE (patient/control ratio:1.9, p<0.0001; 1.7, p<0.0001; 1.7, p<0.0001; 1.6, p<0.0001, respectively). As well, leucylarginine, leucylaspartate, leucylglutamate, leucylthreonine and leucylhistidine were detected with significantly higher amounts in PXE fibroblasts (patient/control ratio:1.9, p<0.005; 1.4, p<0.002; 1.4, p<0.0004; 1.7, p<0.002; 1.8, p<0.002, respectively). However, no significant differences were observed between siRNA-transfected cells, although leucylpeptide levels of ABCC6-silenced fibroblasts were slightly decreased, the difference was not significant (Figure 4).

In addition to leucine peptides, cellular levels of isoleucylleucine, isoleucylthreonine, isoleucylglutamate and isoleucylglutamine were also increased in PXE lysates (Table S2; patient/control ratio:1.3, p<0.03; 1.3, p<0.003; 1.5, p<0.003; 1.4, p<0.002, respectively).

### Elevated levels of arginylproline and polypeptides

**Figure 5:**
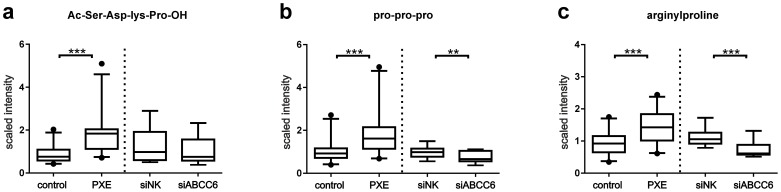
Elevated levels of arginylproline and polypeptides. (a) Amounts of Ac-Ser-Asp-Lys-Pro-OH (AcSDKP; patient/control ratio:, p<0.0001) and proline oligonucleotides (pro-pro-pro) were measured by LC-MS/MS in positive ionization mode. (b) Levels of proline oligonucleotides (pro-pro-pro; patient/control ratio:1.8, p<0.0005; siNK/siABCC6 ratio:0.7, p<0.008) (c) Detection of arginylproline by LC-MS/MS pos. (patient/control ratio:1.5, p<0.0002; siNK/siABCC6 ratio:0.7, p<0.0009).

As shown in Figure 5, two polypeptides and arginylproline were significantly increased in PXE fibroblasts. Ac-Ser-Asp-Lys-Pro-OH (AcSDKP), a polypeptide that is characterized as the N-fragment (1–4) of thymosin beta 4 [27], was 2.1-fold higher than in controls (Figure 5a; patient/control ratio, p<0.0001). Proline oligopeptides (pro-pro-pro), which can be derived from collagen degradation [28] and reflect extracellular matrix remodeling, were found in higher amounts in PXE fibroblasts (Figure 5b; patient/control ratio:1.8, p<0.0005), whereas siRNA-transfected cells showed the opposite effect (Figure 5b; siNK/siABCC6 ratio:0.7, p<0.008). The same trend was also observed for arginylproline with significantly higher amounts in PXE cells (Figure 5c; patient/control ratio:1.5, p<0.0002) and lower levels in ABCC6-silenced cells (Figure 5c; siNK/siABCC6 ratio:0.7, p<0.0009).

### Alterations in pantothenate and CoA metabolism in PXE fibroblasts

**Figure 6:**
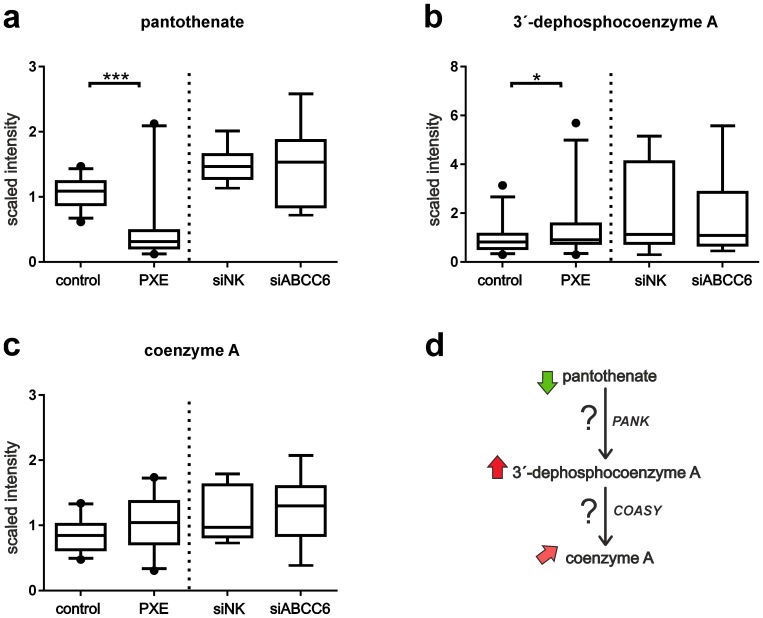
Pantothenate metabolism. (a) Measurements of pantothenate by LC-MS/MS in positive ionization mode (patient/control ratio:0.5, p<0.0003). (b) 3′-dephosphocoenzyme A (patient/control ratio:1.6, p<0.05) and (c) coenzyme A (patient/control ratio:1.2, p = 0.051) were detected by LC-MS/MS negative. No alterations in this metabolic pathway could be observed for siRNA-transfected cells. (d) Metabolic pathway of coenzyme A synthesis from pantothenate, including enzymatic steps of pantothenate kinases (PANK) and CoA synthase (COASY). Arrows point to the comparison of patients and healthy controls: red (significantly increased), light red (increased, by trend), green (significantly decreased).

Fibroblasts from PXE patients possessed significantly lower levels of pantothenate (vitamin B5) (Figure 6a; patient/control ratio:0.5, p<0.0003), an essential metabolite for the synthesis of coenzyme A [29]. Coenzyme A was slightly elevated in PXE samples relative to healthy controls (Figure 6c; patient/control ratio:1.2, p = 0.051), as well as 3′-dephosphocoenzyme A (Figure 6b; patient/control ratio:1.6, p<0.05) as an intermediate in pantothenate and coenzyme A biosynthesis. However, no alterations in this metabolic pathway were observed for siRNA-transfected cells (Figure 6a–c).

### Purine metabolism (Guanine/Adenine/Xanthine/Urate)

**Figure 7:**
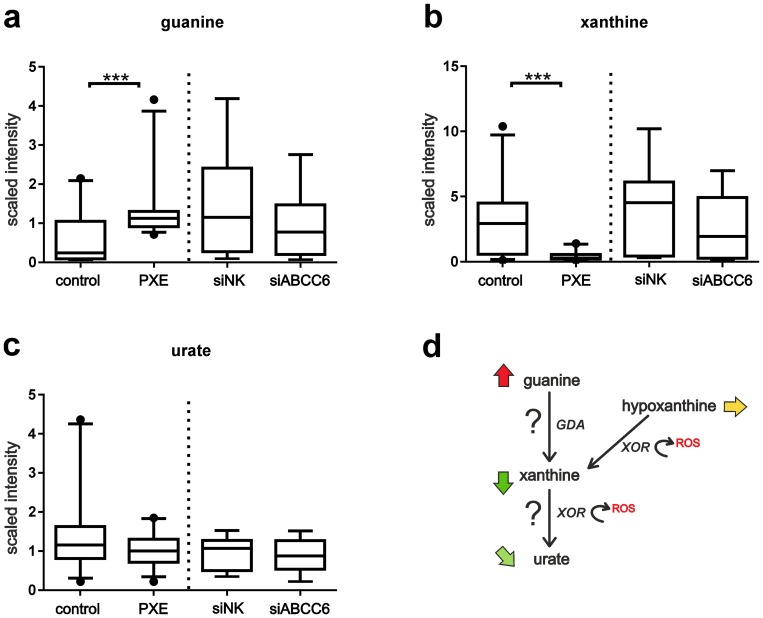
Guanine metabolism. (a) Guanine levels were detected by LC-MS/MS in positive ionization mode (patient/control ratio:2.4, p<0.0001). (b) Xanthines (patient/control ratio:0.1, p<0.0001; siABCC6: siNK ratio 0.6, p = 0.1) and (c) urate levels (patient/control ratio:0.7, p = 0.05) were monitored by GC/MS. (d) Metabolic pathway of purine breakdown of guanine, including enzymatic steps of guanine deaminases (GDA) and xanthine oxidoreductases (XOR) with formation of reactive oxygen species (ROS). Arrows point to the comparison of patients and healthy controls: red (significantly increased), green (significantly decreased), light green (decreased, by trend), yellow (unchanged).

Compared to controls, guanine levels were significantly increased in all PXE cell lines (Figure 7a; patient/control ratio:2.4, p<0.0001). In contrast, related purine nucleosides such as guanosine, 2′-deoxyguanosine, guanosine 5′-monophosphate (5′-GMP) and guanosine 5′-diphospho-fucose did not differ between patients samples (Table S2). Neither adenine, nor the related nucleosides [adenosine, adenosine 2′-monophosphate (2′-AMP), adenosine 3′-monophosphate (3′-AMP), adenosine 5′-monophosphate (AMP), adenosine 5′-diphosphate (ADP) and adenosine 5′-triphosphate (ATP)] were found in higher amounts in PXE fibroblasts (Figure S4). However, significantly lower levels of adenosine 2′-monophosphate (2′-AMP) were detected in siRNA-transfected cells relative to siNK controls (Figure S4 siABCC6/siNK ratio:0.6, p<0.02).

Xanthine, another metabolite in purine metabolism, was strongly diminished in PXE fibroblasts (Figure 7b; patient/control ratio:0.1, p<0.0001) and slightly depleted in ABCC6-silenced cells (Figure 7b; siABCC6/siNK ratio:0.6, p = 0.1). No effects were observed in hypoxanthine or inosine levels (Table S2). Urate (also known as uric acid), another biochemical product of the purine breakdown, was diminished in PXE cell lysates (Figure 7c; patient/control ratio:0.7, p = 0.05), whereas no difference was found between siRNA-transfected fibroblasts.

## Discussion

**Figure 8:**
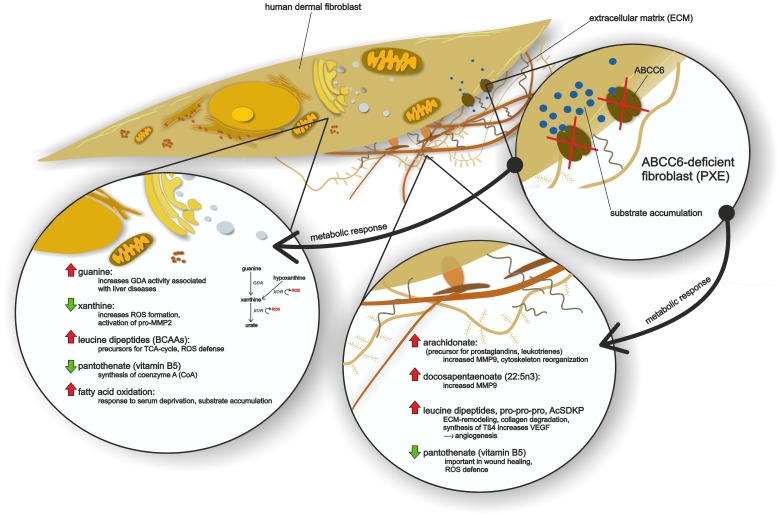
Summary of metabolic findings in PXE human dermal fibroblasts compared to healthy controls.

We describe a metabolomic profiling study showing biochemical alterations in human dermal fibroblasts of PXE patients and ABCC6-silenced cells. The identification of 358 named metabolites allowed for a thorough evaluation of differences in biochemical pathways between PXE patients and controls. The most important findings in this survey are the profound differences in lipid composition, leucine dipeptides, polypeptides, and alterations in pantothenate and purine metabolism, summarized in Figure 8.

Interestingly, metabolic differences between PXE fibroblasts and healthy controls were rarely confirmed in siRNA-transfected cells. Although, ABCC6 knock-down efficiency was proven to be 75%, no reduction in protein level of ABCC6 was observed by Western Blot analysis. One potential explanation for this discrepancy is that the used transfection method with liposomes somehow impacted cellular metabolism. To address this limitation, siRNA-transfected cells were excluded for detailed lipid analysis. It could also be possible that the metabolic alterations observed in PXE fibroblasts derive from metabolic changes which develop on later time points. The effect of ABCC6 knock-down on the metabolome of dermal fibroblasts was analyzed after 72 h, maybe too soon. A cellular model with stable knock-down of ABCC6, also verified on protein level, would be important for follow up studies.

Gene expression analysis for ABCC6 demonstrated that almost no mRNA could be detected in PXE fibroblasts [10]. Analysis by Western Blot showed a clearly lower protein expression of ABCC6 in skin fibroblasts compared to the positive control HEK-293. Surprisingly, about 70% of control protein levels of ABCC6 were also found in PXE fibroblasts. Certainly, one limitation of our study is the varying genetic background of each individual analyzed here. PXE patients 1–6 showed different protein expression levels of ABCC6 (ranging between 28 and 139% of controls) highlighting the individual genetic background of each patient. Some ABCC6 mutations may result in a complete loss of ABCC6 mutations (e.g. from nonsense mutations) due to post-transcriptional or translational regulations, whereas others led to intracellular protein accumulation due to mislocalization/misfolding [30]. Keitel *et al*. showed an accumulation of ABCC2 mutant protein within the endoplasmic reticulum (ER) in patients with *Dubin-Johnson Syndrome* carrying two amino-acid deletions [31], [32].

Interestingly, fibroblasts from PXE patient 2 showing the lowest protein content, were found to carry two heterozygous ABCC6 mutations c.3421C>T (p.R1141X) and c.2787+1G>T. Furthermore, PXE patient 5 with 69% protein level detected, exhibit a genetic variation within the promotor region (c.-90ins14) in addition to the frequently observed c.3421C>T (p.R1141X) mutation. However, no correlations between ABCC6 protein expression and genotypes were described in cells derived from PXE patients, so far. Furthermore, different protein trafficking and cellular accumulation was previously shown for different ABCC6 mutants, especially missense variants, which may also be the case in PXE fibroblasts [33]. However, this is the first study which shows protein detection of ABCC6 in fibroblasts by Western Blot, confirming previously described ABCC6 expression in dermal fibroblasts by immunofluorescence [34]. Due to the known difficulty to obtain material from patients suffering from a rare disease, it was not possible to analyze samples from patients carrying the same type of ABCC6 mutation. Furthermore, the variability in the strength of the metabolic alterations seen for each cell line is unsurprising as each individual presents with not only a clear pathogenic ABCC6 genotype but also a distinct genetic background (a limitation for each biomarker). The significant metabolic alterations found here should therefore be considered as characteristic for a PXE phenotype and assumed to correlate with a functional loss of ABCC6, even though each individual might have a pathogenetically unique ABCC6 genotype.

Functional loss of ABCC6 could lead to a substrate accumulation in the cytosol with impacts on intra- and extracellular metabolism. The “metabolic hypothesis” can be explained by the expression pattern of ABCC6, which is predominantly restricted to the liver. In case of functional loss of ABCC6, circulating levels of unknown substrate/s that might counteract mineralization processes in peripheral tissues like vessels and skin are missing [35]. However, “the peripheral cell hypothesis” adds the fact, that *in vitro* cultivated skin fibroblasts exhibit a characteristic cellular phenotype of PXE significantly different from healthy control cells, which cannot be explained by the expression of ABCC6 in the liver [35]. Omitting serum supplementation for 24 h in our experimental design should enhance metabolic activity of cells and induce oxidative stress, as described for PXE [15]. On the other hand, serum deprivation should lead to an endogenous synthesis and accumulation of newly synthesized metabolites in ABCC6 deficient PXE fibroblasts which might also be possible substrates of ABCC6. Furthermore, newly published data showed significantly induced ABCC6 mRNA expression under serum starvation, relative to standard cultivation in 10% FCS [23].

Altered levels of fatty acids, leucine dipeptides, pro-pro-pro, AcSDKP and pantothenate in cell lysates of PXE patients relative to healthy controls were found. These observations can be linked to extracellular effects, like cytoskeleton and ECM reorganization, atherogenesis and angiogenesis.

Solakivi *et al.* showed a positive correlation between docosapentaenoate (22:5n3) levels and circulating amounts of MMP-9 [36]. They found increased amounts in cases of subclinical inflammation, suggesting a possible response to increased pro-inflammatory cytokine production (tumor necrosis factor alpha and interleukin-1-β) which could be inhibited by anti-inflammatory action of n-3 polyunsaturated fatty acids. Similarly, elevated levels of MMP-9 have previously been reported for PXE serum samples [3].

Glycerophospholipids are essential components of the outer cell membrane, organelles, lipid droplets, or lipoprotein particles [37], [38]. However, products of phospholipid oxidation are suspected of being involved in atherogenesis [39]. Arachidonic acid, generated from phospholipids, is a precursor for different eicosanoids, like prostaglandins and leukotrienes [40], which were described as substrates for ABCC6 and ABCC1 *in vitro* [13], [41]. Elevated levels of arachidonate (20:4n6) increased MMP-9 gene expression and secretion in a dose-dependent manner in human monocytic MonoMac 6 cells [42]. Although arachidonate is normally bound to lipoproteins, albumin, or lysophospholipids, increased cellular levels of free arachidonate are often detected during stress and inflammatory conditions [42]. Additionally, prostaglandins are involved in cytoskeleton reorganization or remodeling [43] and are increased during stress-induced fibroblast senescence [44]. However, levels of prostaglandins 1 and 2 were not elevated in all PXE cells or siRNA silenced fibroblasts.

For clarification, future studies should measure cellular leukotriene levels in PXE cells and siRNA-silenced fibroblasts. Increased levels of branched-chain amino acids (BCAA), predominately leucine dipeptides, indicate a higher energetic demand and oxidative defense in PXE fibroblasts [45]. BCAAs serve as important precursors of branched long chain fatty acids, which have been found in lipids of rat skin and in the cover of the retina [46]. Defects in branched-chain α-ketoacid dehydrogenase complex (BCKDC), which catabolizes BCAAs causes toxically stages like those seen in maple syrup urine disease where increasing amounts of BCAAs leads to neurological dysfunction and brain damage [46].

Extracellular structures like decorin or biglycan are members of the small leucine-rich proteoglycans (SLRPs) which play regulatory roles in collagen fibril growth, fibril organization and extracellular matrix assembly [47]. Recent studies have detected increased biosynthesis, urinary excretion in PXE patients [48], [49] and higher degradation rates for glucosamine-containing GAGs in human dermal fibroblasts derived from PXE patients [50] indicating that proteoglycan synthesis may be dysregulated in PXE patients. Thus, leucine dipeptides, which were found in significantly higher amounts in PXE cell lysates compared to controls, may also be a result of SLRP degradation during matrix reorganization. However, ABCC6-silenced cells exhibit no increase of leucine dipeptides. Furthermore, scaled intensities for these metabolites were overall diminished (but not significantly) in comparison to scrambled siRNA negative controls.

In addition to leucine dipeptides, arginylproline and proline oligopeptides (pro-pro-pro) were found in significantly higher amounts in PXE cells. High levels of proline are stored in collagen of the extracellular matrix, connective tissues and bones [51]. The remodeling of connective fibers like collagen, by MMPs and prolidases, can lead to increased cellular free prolines and hydroxyprolines [51], and proline oxidases (POX) catalyzes the first step of proline degradation in the inner mitochondrial membrane including the formation of reactive oxygen species (ROS) [52].

Scaled intensity of Ac-Ser-Asp-Lys-Pro-OH (AcSDKP), another polypeptide that was analyzed in this study, was 2.1-fold higher in PXE cells than in healthy controls. Moreover, it is believed that AcSDKP is involved in extracellular modeling and in suppressing collagen production by inhibition of TGFß/Smad/ERK1/2 signaling pathway [53]. AcSDKP is characterized as the N-fragment (1–4) of thymosin beta 4 [27], which might potentially be the metabolic precursor for this tetrapeptide [54]. Thymosin beta 4 (Tβ4) regulates osteogenetic factors like the activity of alkaline phosphatase (ALP), calcific nodule formation in human dental pulp cells, and activation of Tβ4 stimulated Runx2 mRNA expression [55]. Boraldi *et al*. observed higher rates of ALP activity in fibroblasts derived from PXE patients relative to controls [56]. Furthermore, Tβ4 and its peptide AcSDKP have also been associated with angiogenesis [54], a hallmark of PXE retinopathy [57]. Single nucleotide variants of the vascular endothelial growth factor (VEGF) have been pinpointed as potential genetic risk factors for ocular manifestations in PXE patients [57]. Jo *et al.* demonstrate that VEGF can be induced by Tβ4 [58], and that increased gene expression of VEGF and Tβ4 were found in the retina of rats with diabetic retinopathy [59]. In this context, high levels of the polypeptide AcSDKP in cell lysates of PXE patients suggest an involvement of thymosin beta 4 in the reorganization of the extracellular matrix in human dermal fibroblasts. Moreover, studies revealed that Tβ4 could be induced by tenascin-C, an extracellular matrix protein involved in bone mineralization and initial valvular calcification [60]. Thus, Tβ4 might be a promising link between ECM remodeling, angiogenesis and vascular calcification in PXE and should be investigated in further studies.

We found significantly reduced levels of pantothenate (vitamin B5) in PXE fibroblasts which had never before been associated with PXE. Compared with healthy control cells, PXE cell lysates had only about the half of the scaled intensity. However, intensities measured for siRNA-transfected cells were clearly higher than for PXE and control cells, perhaps influenced by liposome-based transfection. Pantothenate, a water-soluble B vitamin, is ingested by various foods and is an essential precursor for coenzyme A (CoA), which is needed for metabolism of carbohydrates, proteins and lipids [61]. Pantothenate is known for its beneficial effects in wound healing of skin, especially the alcohol analogue panthenol, and has been shown to reduce the formation of free radicals during oxidative stress [62], [63]. Wiederholt *et al.* investigated the effects of pantothenate supplementation on gene expression patterns of human dermal fibroblasts, showing increased expression levels for interleukins and other proteins regulated by oxidation events [63]. Pantothenate is a precursor for cellular coenzyme A (CoA) synthesis, which is conducted by pantothenate kinases (PANK) [64]. Amounts of coenzyme A were slightly increased in PXE fibroblasts. Moreover, significantly increased levels were measured for its precursor 3′-dephosphocoenzyme A. Most cellular CoA is located in mitochondria, where it can be regenerated from acetyl-CoA, ketone body synthesis or fatty acid-CoA [65]. A minor part is stored and degraded in peroxisomes or the cytosol [65]. A higher demand on CoA, e.g. for mitochondrial ß-oxidation, could explain an increased turnover of pantothenate, leading to reduced pantothenate levels as found in PXE cells. These intracellular effects of decreased pantothenate levels in PXE fibroblasts can be add by alterations in guanine and xanthine metabolism. Recent studies revealed that adenosine, suspected to be involved in pathogenesis of PXE, is not transported by ABCC6 in Sf9 insect cell transport systems [66]. Similar, in this study no differences of adenine and its nucleosides were found between PXE and healthy control fibroblasts. Furthermore, in contrast to the study published by Jansen *et al.* [20] where increased nucleotide/nucleoside concentrations in ABCC6-conditioned media and low inorganic pyrophosphate plasma concentrations in Abcc6 ^−/−^ mice were found, no significant differences were found in nucleoside triphosphate, nor in pyrophosphate (PPi) levels between PXE and control groups in this study. Lower levels of adenosine 2′-monophosphate (2′-AMP) were only detected in siRNA-transfected cells compared to siRNA negative controls. Guanine was the metabolite with the largest difference in concentration between PXE and healthy fibroblasts. Samples derived from PXE cells exhibit 2.4-fold higher levels of guanine, while ABCC6-silenced cells showed slightly lower guanine amounts compared to siNK controls. Higher guanosine phosphate (e.g. GTP) turnover could enhance cellular pyrophosphate supply [20], while simultaneously increasing the intracellular level of guanine. Pyrophosphate, as a major calcification inhibitor, and pyrophosphate metabolizing enzymes were investigated in newly published studies concerning PXE pathophysiology [67]–[69]. Interestingly, both *de novo* syntheses of purine nucleotides and recycling of purines through *salvage pathway* leads to cellular pyrophosphate release [70]. Hence, a higher recycling rate of guanine instead of excretion through uric acid could enhance cellular supply of pyrophosphates in PXE fibroblasts. Interestingly, xanthine was significantly reduced in PXE cell lysates, and slightly depleted in siRNA-transfected cells. Xanthines are catabolized from guanine by guanine deaminases (GDA), and higher levels of GDA activity were associated with liver diseases, like chronic hepatitis, biliary obstruction, and liver cirrhosis [71]. Xanthines are further metabolized to urate/uric acid by xanthine oxidoreductases (XOR), and ROS produced by oxidases are involved in atherosclerotic progressions [72]. Increased purine recycling by the cellular *salvage pathway* could decrease XOR activity in PXE fibroblasts and protect from additional oxidative stress.

Largest activities of xanthine oxidoreductases are found in the liver and intestine [73], which matches the tissue specific expression pattern of ABCC6. Scaled intensities of urate were also slightly diminished in PXE and ABCC6-silenced cells. Antioxidative properties were described for urate by Becker *et al.* [74], and alterations of purine catabolism are described as a homeostatic response of mitochondria to oxidative stress [75]. Therefore, decreased levels of xanthines in PXE fibroblasts could be due to increased XOR activities, or impaired activities of the guanine catabolizing enzyme GDA. The latter might be a further explanation for the elevated amounts of guanine found in PXE cell lysates. However, regulating mechanisms of GDA should be examined in future studies.

## Perspectives

In summary, this untargeted metabolic approach revealed significant differences in lipid composition, leucine dipeptides, polypeptides, and alterations in pantothenate and guanine metabolism in PXE. Our findings could be linked to increased extracellular matrix remodeling, oxidative stress and enhanced fatty acid oxidation in PXE cells. High levels of the polypeptide AcSDKP in cell lysates of PXE patients suggest an involvement of thymosin beta 4 (Tβ4) in the reorganization of the extracellular matrix. In this context, Tβ4 and tenascin-C might be important regulators in ECM remodeling, angiogenesis and vascular calcification in PXE. Dysregulations in pantothenate (vitamin B5) and xanthine/guanine metabolism were additional key findings of our experiments. These results should be further proven, e.g. by monitoring oxidative stress level or matrix degradation after the addition of pantothenate to cell culture settings. Specifically, gene and protein expression analysis of targets involved in pantothenate and xanthine/guanine metabolism (e.g. PANK, GDA and XOR) should be conducted to confirm the results of this study. This large metabolic profiling of cell lysates from PXE patients and controls could lead to a greater understanding of biochemical pathways involved in soft tissue mineralization, which can be potentially used to explore general questions regarding related human etiopathologies.

## Supporting Information

Figure S1 **Principal component analysis (PCA).**
(TIF)

Figure S2 **Fatty acids.** Essential fatty acids, like (a) dihomo-linolenate (20:3n3 or n6; patient/control ratio:1.4, p<0.03), (b) docosapentaenoate (22:5n3; patient/control ratio:1.5, p<0.0006) and (c) docosapentaenoate (22:5n6; patient control ratio 1.3, p<0.05) were detected by LC-MS/MS in negative ionization mode. Long chain fatty acids (d) Arachidonate (20:4n6; patient/control ratio:1.5, p<0.005) and (e) adrenate (22:4n6; patient/control ratio:1.3, p<0.04) were detected by LC-MS/MS neg. (f) Amounts of monohydroxy fatty acid 4-hydroxybutyrate (GHB; patient/control ratio:1.5, p<0.001) were measured by GC/MS.
(TIF)

Figure S3 **Sphingolipids, Sterols.** Levels of (a) sphingosine (patient/control ratio:1.3, p<0.02) and (b) palmitoyl sphingomyelin (patient/control ratio:1.3, p<0.0001) were detected by LC-MS/MS positive and GC/MS, respectively. (c) The amounts of lathosterol (patient/control ratio:1.4, p<0.05) were measured by GC/MS.
(TIF)

Figure S4 **Adenine metabolism.** Biochemical levels of (a) adenine, (b) adenosine, (c) adenosine 2′-monophosphate (2′-AMP), (d) adenosine 3′-monophosphate (3′-AMP), (e) adenosine 5′-monophosphate (AMP), (f) adenosine 5′-diphosphate (ADP) and (g) adenosine 5′-triphosphate (ATP)] were not significantly different in PXE fibroblasts. Significantly lower levels of (c) adenosine 2′-monophosphate (2′-AMP) were detected in siRNA-transfected cells compared to FAM labeled controls (siABCC6: siNK ratio 0.6, p<0.02).
(TIF)

Table S1 **Characterization NHDFs.**
(PDF)

Table S2 **Heat map: results metabolic profiling.**
(XLSX)

Methods S1 **Detailed description of mass spectrometry analysis.**
(DOCX)
