# Supplementary figures and images for: Large-Scaled Metabolic Profiling of Human Dermal Fibroblasts Derived from Pseudoxanthoma Elasticum Patients and Healthy Controls
Source: PLoS One. 2014 Sep 29;9(9):e108336. doi: 10.1371/journal.pone.0108336 (PMC4181624; doi:10.1371/journal.pone.0108336)

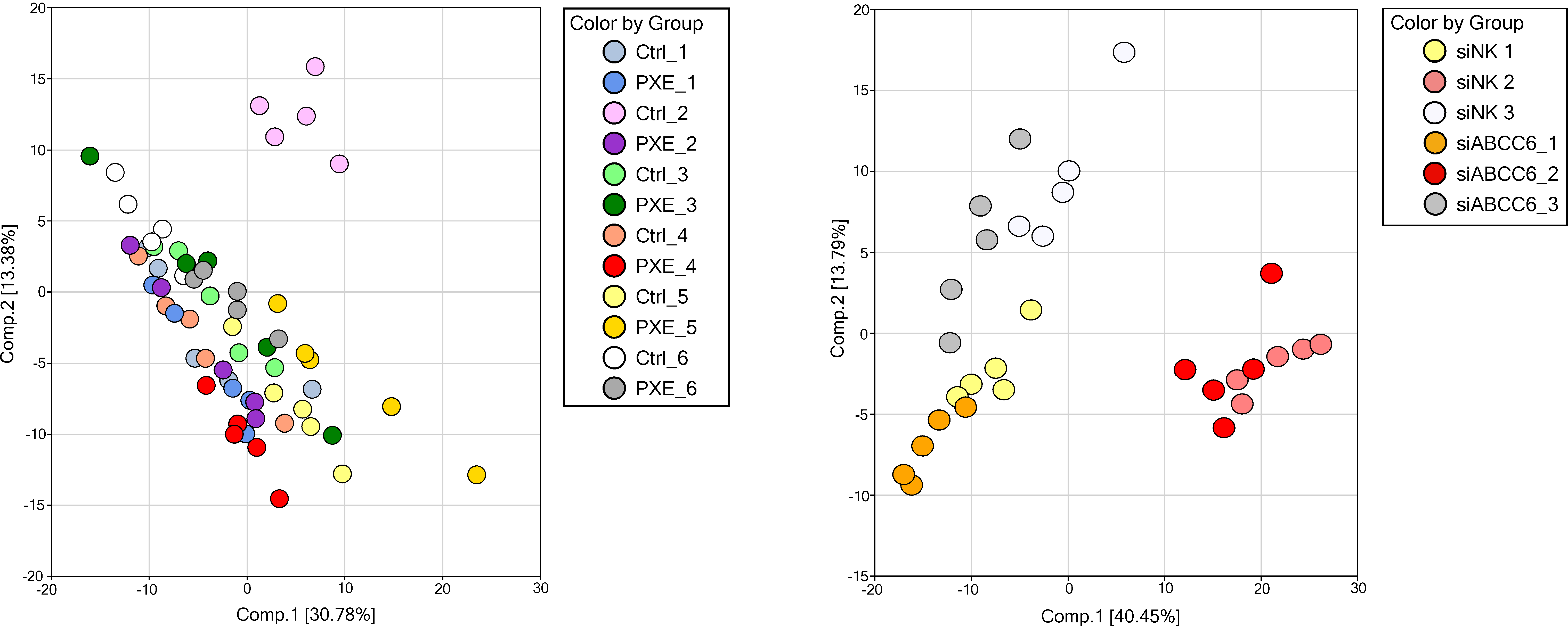

Supplement: Figure S1 — Principal component analysis (PCA). (TIF) [file pone.0108336.s001.tif]

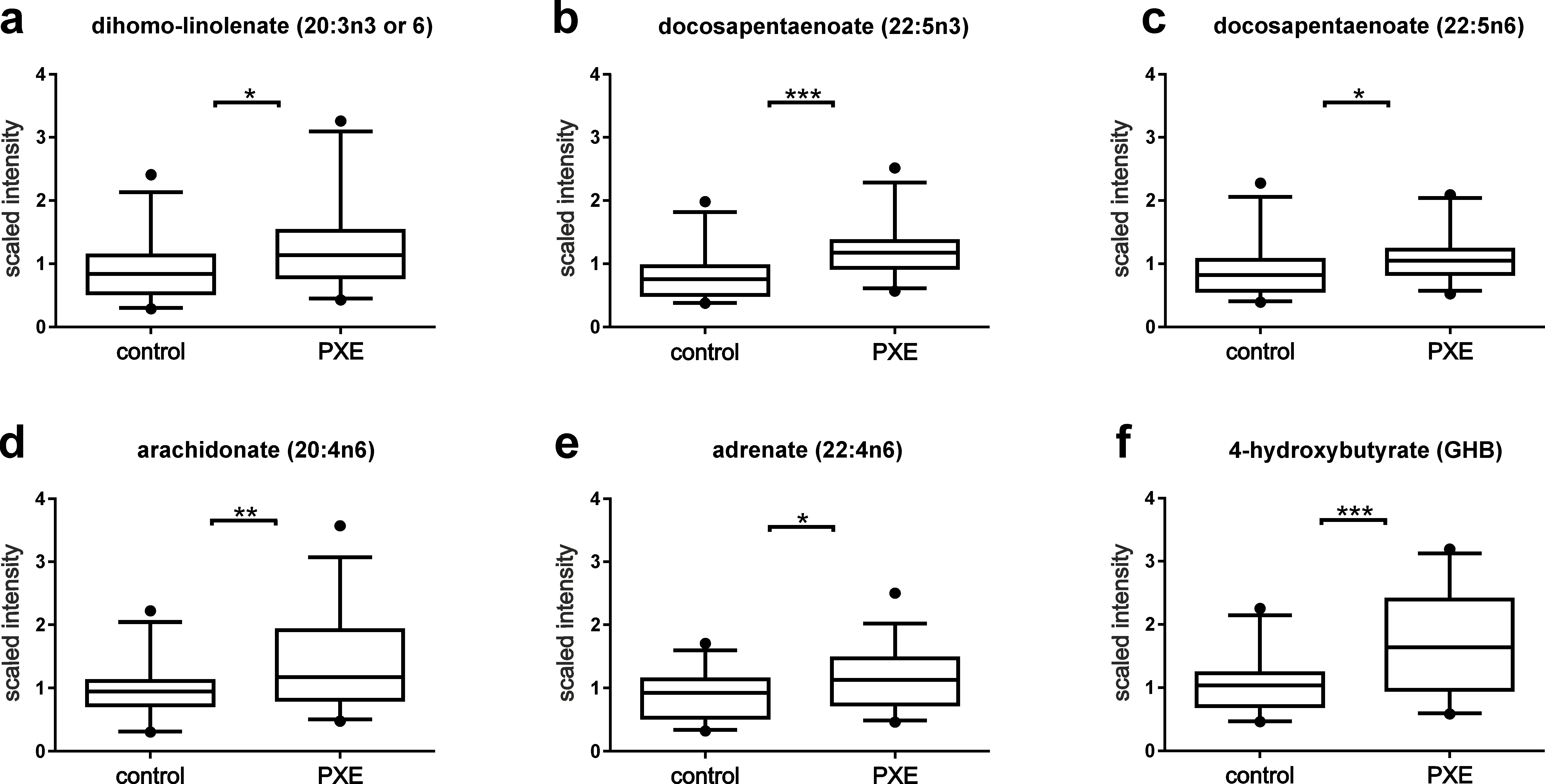

Supplement: Figure S2 — Fatty acids. Essential fatty acids, like (a) dihomo-linolenate (20:3n3 or n6; patient/control ratio:1.4, p<0.03), (b) docosapentaenoate (22:5n3; patient/control ratio:1.5, p<0.0006) and (c) docosapentaenoate (22:5n6; patient control ratio 1.3, p<0.05) were detected by LC-MS/MS in negative ionization mode. Long chain fatty acids (d) Arachidonate (20:4n6; patient/control ratio:1.5, p<0.005) and (e) adrenate (22:4n6; patient/control ratio:1.3, p<0.04) were detected by LC-MS/MS neg. (f) Amounts of monohydroxy fatty acid 4-hydroxybutyrate (GHB; patient/control ratio:1.5, p<0.001) were measured by GC/MS. (TIF) [file pone.0108336.s002.tif]

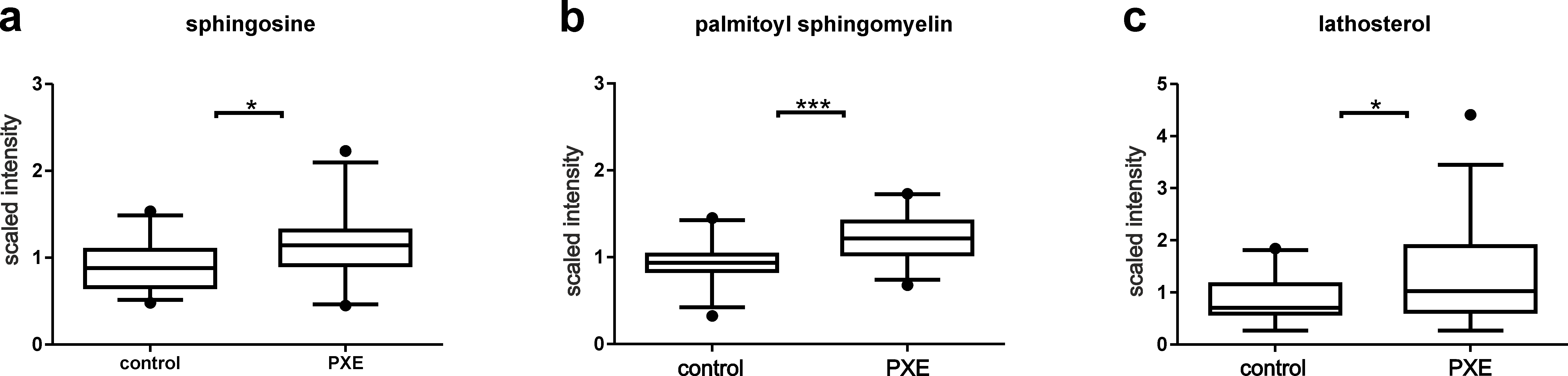

Supplement: Figure S3 — Sphingolipids, Sterols. Levels of (a) sphingosine (patient/control ratio:1.3, p<0.02) and (b) palmitoyl sphingomyelin (patient/control ratio:1.3, p<0.0001) were detected by LC-MS/MS positive and GC/MS, respectively. (c) The amounts of lathosterol (patient/control ratio:1.4, p<0.05) were measured by GC/MS. (TIF) [file pone.0108336.s003.tif]

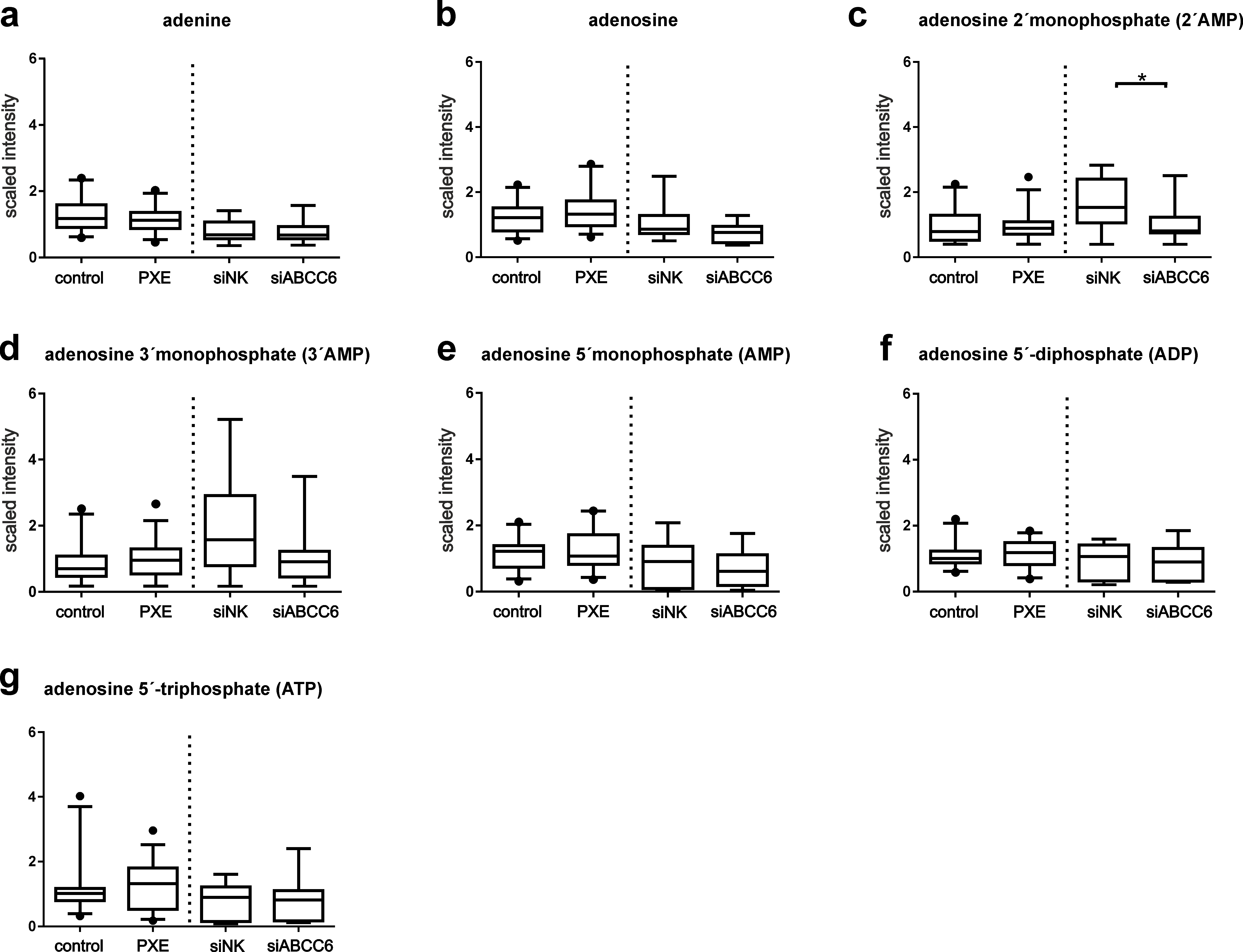

Supplement: Figure S4 — Adenine metabolism. Biochemical levels of (a) adenine, (b) adenosine, (c) adenosine 2′-monophosphate (2′-AMP), (d) adenosine 3′-monophosphate (3′-AMP), (e) adenosine 5′-monophosphate (AMP), (f) adenosine 5′-diphosphate (ADP) and (g) adenosine 5′-triphosphate (ATP)] were not significantly different in PXE fibroblasts. Significantly lower levels of (c) adenosine 2′-monophosphate (2′-AMP) were detected in siRNA-transfected cells compared to FAM labeled controls (siABCC6: siNK ratio 0.6, p<0.02). (TIF) [file pone.0108336.s004.tif]
