# Supplementary material for: Large-Scaled Metabolic Profiling of Human Dermal Fibroblasts Derived from Pseudoxanthoma Elasticum Patients and Healthy Controls
Source: PLoS One. 2014 Sep 29;9(9):e108336. doi: 10.1371/journal.pone.0108336 (PMC4181624; doi:10.1371/journal.pone.0108336)
Supplement: Table S1 — Characterization NHDFs. (PDF) [file pone.0108336.s005.pdf]

Sample iD	Catalog ID	Source	Gender	Age (years)	Biopsy source	*ABCC6* genotype[a]		Genotype status
**PXE patients**								
PXE 1		*	male	51	Neck	c.3769_3770insC (p.L1259fsX1277)	c.3769_3770insC (p.L1259fsX1277)	hm
PXE 2		*	female	n/a	Armpit	c.3421C4T (p.R1141X)	c.2787+1G>T	cht
PXE 3		*	male	41	n/a	c.1552C4T (p.R518X)	n/d	ht
PXE 4		*	female	62	Neck	c.1132C4T (p.Q378X)	c.3421C4T (p.R1141X)	cht
PXE 5		*	male	42	n/a	c.3421C4T (p.R1141X)	c.-90ins14	(c)ht
PXE 6	GM04994	Coriell Institute	male	60	n/a	c.3421C4T (p.R1141X)	c.3490C4T (p.R1164X)	cht
**healthy controls**								
Ctl 1	CC-2511	Cambrex	female	42	Abdomen	-	-	wt
Ctl 2	PH10605A	Genlantis	male	56	Face	-	-	wt
Ctl 3	C-12302	Promocell	female	52	Cheek	-	-	wt
Ctl 4	GM23251	Coriell Institute	female	50	Arm	-	-	wt
Ctl 5	GM23248	Coriell Institute	male	55	Arm	-	-	wt
Ctl 6	GM23250	Coriell Institute	male	44	Arm	-	-	wt
**siRNA-treated fibroblasts (siNK/ siABCC6)**								
siNK/ ABCC6_1	C-12302	Promocell	female	52	Cheek	-	-	wt
siNK/ ABCC6_2	PH10605A	Genlantis	male	56	Face	-	-	wt
siNK/ ABCC6_3	CC-2511	Cambrex	female	42	Abdomen	-	-	wt

hm, homozygote; cht, compound heterozygote; ht, heterozygote; wt, wild type; n/a, not applicable; n/d, not detected.

* fibroblasts isolated from skin biopsies (Hendig et al. 2008, *Lab. Invest.*)

[a] Nucleotide numbering refers to the cDNA sequence with the A of the ATG translation initiation start site as nucleotide +1 (GenBank accession number NM_001171.2)
