# Supplementary material for: Large-Scaled Metabolic Profiling of Human Dermal Fibroblasts Derived from Pseudoxanthoma Elasticum Patients and Healthy Controls
Source: PLoS One. 2014 Sep 29;9(9):e108336. doi: 10.1371/journal.pone.0108336 (PMC4181624; doi:10.1371/journal.pone.0108336)
Supplement: Methods S1 — Detailed description of mass spectrometry analysis. (DOCX) [file pone.0108336.s007.docx]

*Sample Accessioning*

Each sample received was accessioned into the Metabolon LIMS system and was assigned by the LIMS a unique identifier that was associated with the original source identifier only. This identifier was used to track all sample handling, tasks, results etc. The samples (and all derived aliquots) were tracked by the LIMS system. All portions of any sample were automatically assigned their own unique identifiers by the LIMS when a new task is created; the relationship of these samples is also tracked. All samples were maintained at -80 ºC until processed.

*Sample Preparation*

Samples were prepared using the automated MicroLab STAR® system from Hamilton Company. A recovery standard was added prior to the first step in the extraction process for QC purposes. Sample preparation was conducted using aqueous methanol extraction process to remove the protein fraction while allowing maximum recovery of small molecules. The resulting extract was divided into four fractions: one for analysis by UPLC/MS/MS (positive mode), one for UPLC/MS/MS (negative mode), one for GC/MS, and one for backup. Samples were placed briefly on a TurboVap® (Zymark) to remove the organic solvent. Each sample was then frozen and dried under vacuum. Samples were then prepared for the appropriate instrument, either UPLC/MS/MS or GC/MS.

*Ultrahigh performance liquid chromatography/Mass Spectroscopy (UPLC/MS/MS)*

The LC/MS portion of the platform was based on a Waters ACQUITY ultra-performance liquid chromatography (UPLC) and a Thermo-Finnigan linear trap quadrupole (LTQ) mass spectrometer, which consisted of an electrospray ionization (ESI) source and linear ion-trap (LIT) mass analyzer. The sample extract was dried then reconstituted in acidic or basic LC-compatible solvents, each of which contained 8 or more injection standards at fixed concentrations to ensure injection and chromatographic consistency. One aliquot was analyzed using acidic positive ion optimized conditions and the other using basic negative ion optimized conditions in two independent injections using separate dedicated columns. Extracts reconstituted in acidic conditions were gradient eluted using water and methanol containing 0.1% formic acid, while the basic extracts, which also used water/methanol, contained 6.5mM Ammonium Bicarbonate. The MS analysis alternated between MS and data-dependent MS2 scans using dynamic exclusion. Raw data files are archived and extracted as described below.

*Gas chromatography/Mass Spectroscopy (GC/MS)*

The samples destined for GC/MS analysis were re-dried under vacuum desiccation for a minimum of 24 hours prior to being derivatized under dried nitrogen using bistrimethyl-silyl-triflouroacetamide (BSTFA). The GC column was 5% phenyl and the temperature ramp was from 40° to 300° C in a 16 minute period. Samples were analyzed on a Thermo-Finnigan Trace DSQ fast-scanning single-quadrupole mass spectrometer using electron impact ionization. The instrument was tuned and calibrated for mass resolution and mass accuracy on a daily basis. The information output from the raw data files was automatically extracted as discussed below.

*Quality assurance/QC*

For QA/QC purposes, additional samples were included with each day’s analysis. These samples included extracts of a pool created from a small aliquot of the experimental samples and process blanks. QC samples were spaced evenly among the injections and all experimental samples were randomly distributed throughout the run. A selection of QC compounds was added to every sample for chromatographic alignment, including those under test. These compounds were carefully chosen so as not to interfere with the measurement of the endogenous compounds.

*Data extraction and compound identification*

Raw data was extracted, peak-identified and QC processed using Metabolon’s hardware and software. These systems are built on a web-service platform utilizing Microsoft’s .NET technologies, which run on high-performance application servers and fiber-channel storage arrays in clusters to provide active failover and load-balancing. Compounds were identified by comparison to library entries of purified standards or recurrent unknown entities. More than 3000 commercially available purified standard compounds have been acquired and registered into LIMS for distribution to both the LC and GC platforms for determination of their analytical characteristics.

Statistical Analysis: Missing values (if any) are assumed to be below the level of detection. However, biochemicals that were detected in all samples from one or more groups, but not in samples from other groups were assumed to be near the lower limit of detection in the groups in which they were not detected. In this case, the lowest detected level of these biochemicals was imputed for samples in which that biochemical was not detected. Following log transformation and imputation with minimum observed values for each compound, a Welch’s two-sample t-test and was used to identify biochemicals that differed significantly between experimental groups. Pathways were assigned for each metabolite, allowing examination of overrepresented pathways.

*Statistical analysis*

Missing values (if any) are assumed to be below the level of detection. However, biochemicals that were detected in all samples from one or more groups, but not in samples from other groups were assumed to be near the lower limit of detection in the groups in which they were not detected. In this case, the lowest detected level of these biochemicals was imputed for samples in which that biochemical was not detected. Following log transformation and imputation with minimum observed values for each compound, a Welch’s two-sample t-test and was used to identify biochemicals that differed significantly between experimental groups.
